# Image guidance using 3D-ultrasound (3D-US) for daily positioning of lumpectomy cavity for boost irradiation

**DOI:** 10.1186/1748-717X-6-45

**Published:** 2011-05-09

**Authors:** Manjeet Chadha, Amy Young, Charles Geraghty, Robert Masino, Louis Harrison

**Affiliations:** 1 Department of Radiation Oncology. Beth Israel Medical Center, New York, NY, USA

**Keywords:** breast cancer, electron boost, photon boost, ultrasound, image-guided radiation therapy

## Abstract

**Purpose:**

The goal of this study was to evaluate the use of 3D ultrasound (3DUS) breast IGRT for electron and photon lumpectomy site boost treatments.

**Materials and methods:**

20 patients with a prescribed photon or electron boost were enrolled in this study. 3DUS images were acquired both at time of simulation, to form a coregistered CT/3DUS dataset, and at the time of daily treatment delivery. Intrafractional motion between treatment and simulation 3DUS datasets were calculated to determine IGRT shifts. Photon shifts were evaluated isocentrically, while electron shifts were evaluated in the beam's-eye-view. Volume differences between simulation and first boost fraction were calculated. Further, to control for the effect of change in seroma/cavity volume due to time lapse between the 2 sets of images, interfraction IGRT shifts using the first boost fraction as reference for all subsequent treatment fractions were also calculated.

**Results:**

For photon boosts, IGRT shifts were 1.1 ± 0.5 cm and 50% of fractions required a shift >1.0 cm. Volume change between simulation and boost was 49 ± 31%. Shifts when using the first boost fraction as reference were 0.8 ± 0.4 cm and 24% required a shift >1.0 cm. For electron boosts, shifts were 1.0 ± 0.5 cm and 52% fell outside the dosimetric penumbra. Interfraction analysis relative to the first fraction noted the shifts to be 0.8 ± 0.4 cm and 36% fell outside the penumbra.

**Conclusion:**

The lumpectomy cavity can shift significantly during fractionated radiation therapy. 3DUS can be used to image the cavity and correct for interfractional motion. Further studies to better define the protocol for clinical application of IGRT in breast cancer is needed.

## Introduction

Image-guided radiation therapy (IGRT) is widely accepted as a procedure to correct for interfractional target motion. In treating prostate cancer, for example, IGRT is used to correct the daily shifts in target caused by bladder and rectal filling. The various technologies used for IGRT include surface cameras, tracking fiducials with either x-rays [1-3] or electromagnetic beacons [4,5], CBCT in the treatment room, and 3D ultrasound (3DUS) [6-8].

The clinical value of IGRT in the treatment of breast cancer still needs to be defined [9-11]. There may be shifts in the breast tumor bed from its planned position due to patient setup, breast edema, temporal changes in the cavity and breast anatomy from postoperative recovery, and respiratory motion [12]. Application of IGRT would give us real-time information on interfractional target motion and improve accuracy of beam delivery.

IGRT using cone beam CT is associated with increased radiation exposure to the patient, which is of significant concern among the breast cancer patient population. In exploring non-ionizing IGRT options, the Clarity™ 3DUS System (Resonant Medical, Montreal, Canada) has the advantage in that its daily utilization does not result in excess radiation exposure. Another advantage is that most patients are familiar with this modality as part of the breast cancer diagnostic work up and readily accept the procedure. Furthermore, 3DUS imaging of the breast lumpectomy cavity appears to be an ideal target-specific technique.

Studies evaluating treatment plans of electron boost based on the scar location, pre-operative mammograms, and the operative report have shown significant potential for missing the lumpectomy cavity [13-16]. The routine use of 3-dimensional treatment planning, CT images provide a more complete visualization of the lumpectomy site and are used for target definition. Further, it has illustrated that breast density and cavity size, which sometimes limits cavity visualization on CT images, do not compromise visualization of the target when 3DUS is used. Fusion of CT and 3DUS have been shown to provide complementary information for defining the target [10,17,18].

The rationale for IGRT in breast cancer is based on the fact that delivering a higher dose to the lumpectomy cavity (boost) has shown to improve local control. It is also recognized that clinicians tend to use generous boost volume so as to decrease daily set up errors. Large volumes treated to high dose also are reported to result in inferior outcomes. With use of IGRT in treatment of breast cancer there may be better targeting of tissues at risk while reducing the volume of normal breast tissue being irradiated. The objectives of this initial pilot study were to evaluate the feasibility of adding a 3DUS IGRT procedure in the therapy room to reproducibly acquire quality images of the lumpectomy site, to record the interfractional shifts needed to correct for boost target motion, and establish a role for routine clinical application of IGRT in the treatment of breast cancer.

## Methods and materials

### Patients

Patients were enrolled in an Institutional Review Board approved prospective study. The study goal was to acquire data on 20 patients undergoing breast radiotherapy with or without regional lymph node irradiation following lumpectomy; the patients were split between those receiving photon and electron boost treatments.

### The Clarity Breast System

**Figure 1:**
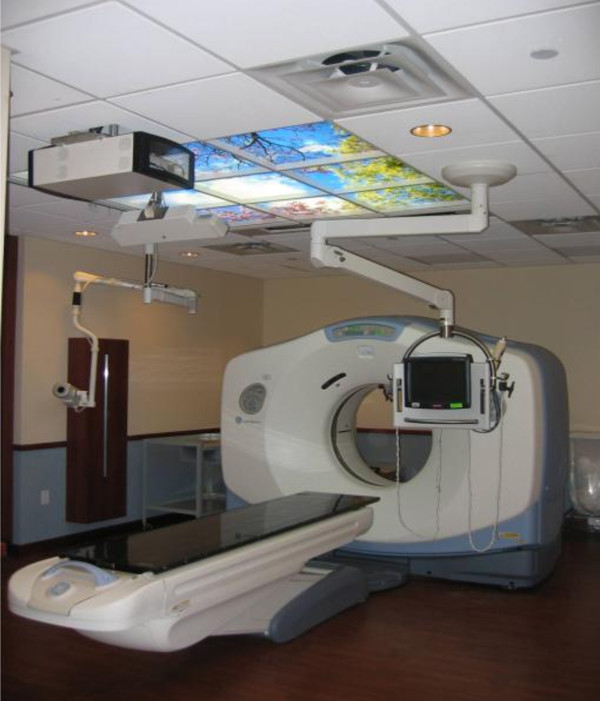
**Clarity system: (a) US-Sim in CT room, and (b) US-Guide in treatment room**.

The Clarity System consists of two 3D-US devices, the US-Sim™ and the US-Guide™, as represented in Figure 1. The US-Sim resides in the CT-Simulation room, whereas the U/S-Guide is in the treatment room; 3D-US images are acquired by scanning the region of interest with an ultrasound probe that has infrared reflective markers affixed to its handle. The markers are tracked by an infrared camera to determine the position and orientation of each ultrasound frame. The frames are then reconstructed to form a 3D voxel dataset. These 3D-US images are calibrated to the room coordinate system of the corresponding CT and treatment room to allow a direct comparison of the reference 3D-US images at simulation to those acquired in the treatment room. This set up allows the same image modality to be used for the comparison. The Clarity breast module uses a high frequency linear probe (central frequency 8 MHz) which allows for a resolution on the order of 0.2 mm.

### Ultrasound Scanning

**Figure 2:**
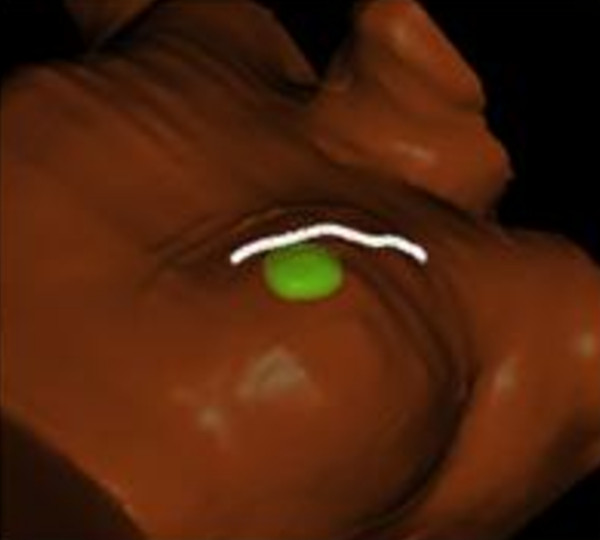
**The Clarity Ultrapath feature, illustrating the scanning path of the probe used at the initial simulation**.

The scanning technique requires a sweep over the area of interest with negligible probe pressure through a thick layer of high viscosity gel [18]. In addition, the *Ultrapath *feature within Clarity illustrates the scanning path of the probe used at the initial simulation as a reference that facilitates reproducibility of image capture on each treatment day. Figure 2.

### CT/3DUS Simulation

The standard procedure for CT simulation was followed. Patients were positioned supine with the ipsilateral arm over head. For immobilization, alpha cradle and breast board were used. Both the CT simulation and 3D-US images were acquired in rapid succession. The CT and 3D-US images were implicitly registered since they shared the same coordinate system through calibration. The registration of the fused CT and 3D-US images was verified qualitatively.

### Treatment planning

**Figure 3:**
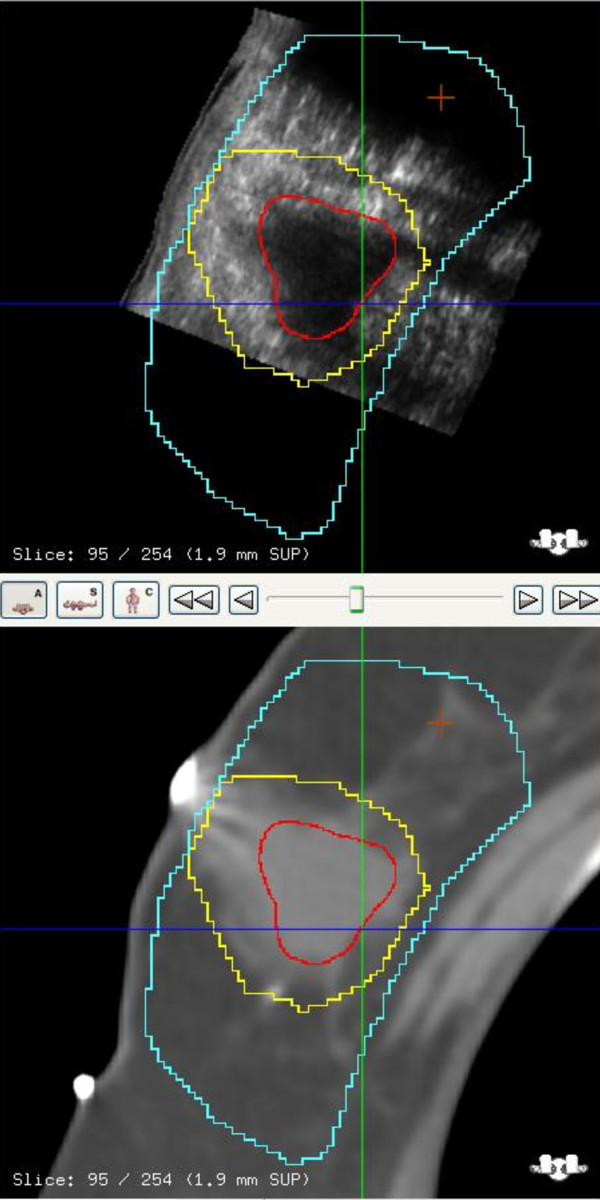
**Axial view of a fused CT/3D-US dataset**. The ultrasound-based reference volume (RV) is in red, the CT-based GTV in yellow, and the PTV in blue.

CT images acquired with 2.5 mm slices from the neck to beyond the inframammary fold were used for defining the various target structures. We use the following definitions in reporting this study: The breast planning volume was defined as the volume of the palpable breast identified on CT. The lumpectomy cavity contoured only on the images from CT simulation identified by both clips and seroma defined the gross target volume (GTV). The boost planning target volume (PTV) was defined as the GTV (lumpectomy cavity) with a 1.5 to 2 cm margin except when there is proximity to overlying skin and underlying chest wall. The seroma contoured on the 3DUS image obtained at simulation served as the *reference volume *(RV) for IGRT. The seroma contoured on 3D-US images acquired on the treatment table during daily therapy is defined as the *guidance volume *(GV) for IGRT. An example of a fused image dataset with both GTV and RV is shown in Figure 3.

For this study, we followed the standard procedure of using only CT simulation images for contouring target volume and normal anatomy. The 3D-US data was not used to modify the contours. Whole-breast radiation therapy was planned using field-in-field forward-planning intensity modulated radiation therapy technique. For the boost plan the choice of using photons or electrons was based on the beam characteristic that delivered an optimal coverage of the boost target volume.

The information of the approved treatment plan isocenter, radiation fields and the CT images were imported into the Clarity Workstation through DICOM transfer. This information was linked to the RV on the 3D-US images from simulation.

### Data acquisition and data analysis

The alignment software in the Clarity system is different for photon and electron boost patients, and thus the technique for data acquisition differed depending on the choice of beam for boost treatments.

**Figure 4:**
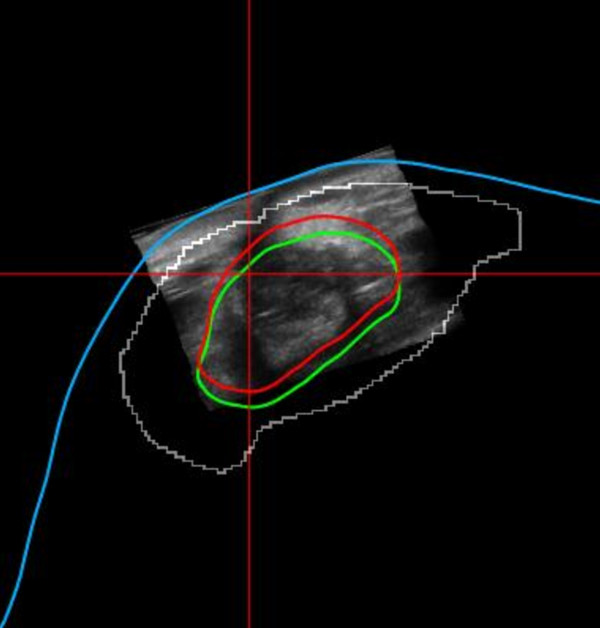
**The RV (green), the GV (red), and the PTV (white)**. The change between the GV and RV indicates the change in seroma positioning between simulation and treatment.

For photon boost, after the patient was positioned in the therapy room 3D-US images were acquired just prior to treatment. The seroma cavity as seen on the 3D-US was contoured using semi-automatic tools on the US-Guide to define the GV for IGRT. The GV was then visualized on the monitor in relation to the RV and PTV, as shown in Figure 4. Calculation of a couch shift required for either aligning the GV to the RV and/or ensuring that the GV falls completely within the PTV of the treatment plan is performed. The couch shifts required for aligning could then be executed by affixing the Clarity couch positioning indicator (CPI), which is tracked by the optical camera in the room coordinate system, to the treatment couch. These shifts were not executed in this study.

**Figure 5:**
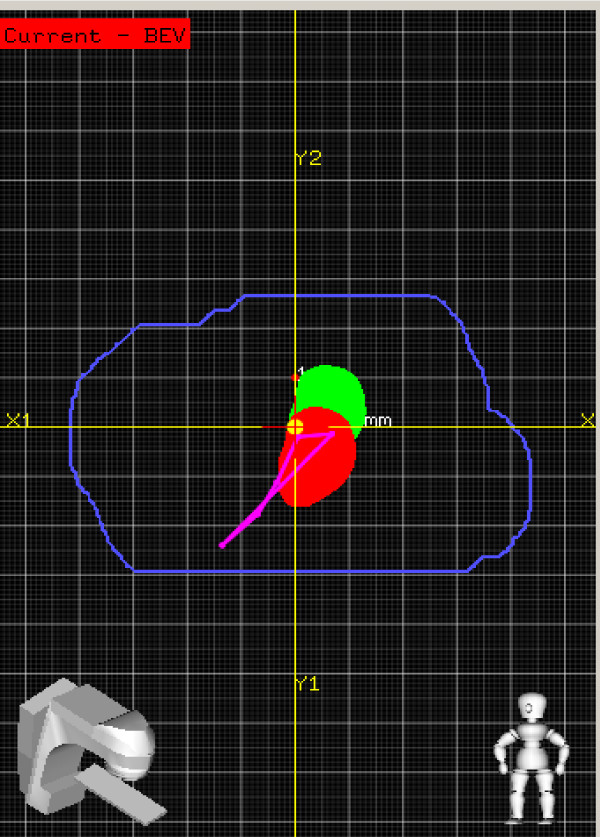
**The GV (red) position versus RV (green) contour of electron boost in the EBEV relative to the cut-out (blue)**. Magenta is the digitized scar.

For electron boosts, patient were aligned according to the instructions of the plan set up maintaining the couch and gantry at zero degrees. The 3D-US image was acquired. The seroma cavity as seen on this image was contoured using semi-automatic tools on the US-Guide to define the GV. The Clarity digitizer, a ball-point tip tool with infrared markers, was used to digitize the scar. This provided both internal and external anatomy in the electron beam's eye view (EBEV). The Clarity couch positioning indicator (CPI) was then affixed to the treatment couch, and the couch was moved and rotated into final treatment position. The CPI tracked these couch motions, and gantry angle changes were typed in manually. As shown in Figure 5, the Clarity screen showed the alignment of the GV and scar relative to the cut-out in real-time as the patient was brought into treatment position, as well as the overlay of the RV reference contour for comparison.

The registration of the fused CT and 3D-US images was verified qualitatively for cavity visibility on both. The overlay of GTV on CT simulation and RV on 3D-US obtained at simulation provided an opportunity to correlating observations between imaging modalities. Further, comparing the RV from the 3D-US obtained at simulation to the GV from the 3D-US obtained at the first treatment fraction provided an opportunity to evaluate the percent change in volume of the seroma cavity.

For photon boosts, we evaluated two potential types of shifts between the GV and RV structures: a) the center-to-center shift between the GV and RV, which would center the cavity at time of treatment; and (b) the center-to-center shift between the GV obtained at the time of the first fraction (which was associated with verification of patient position using port films) with the GV from all remaining treatment fractions. This second method excludes the reference of shifts to simulation so as to minimize the confounding variable of change in size of the seroma between simulation and start of radiation therapy.

For electron boosts, the center-to-center displacement between the GV and RV in the EBEV plane was calculated for each fraction. These were compared to the distance of the cavity to the electron cut-out, minus a margin to account for the known electron dose fall-off for the given electron energy at depth. The displacements were also calculated between the center of the GV for subsequent fractions to the GV obtained at the first fraction of boost treatment.

## Results

Ultrasound IGRT depends on presence of a seroma. Among the screened patients we observed presence of seroma in 93% of cases. Among the 20 patients with seroma enrolled in the study, data on 127 fractions was collected. Data on 14 total fractions, of which 7 were from a single patient, were not evaluable due to systematic errors made while capturing the data. This represented a learning curve for our team and an overall QA compliance of 89% of all fractions studied. Data on the remaining 113 fractions (75 photon boost and 38 electron boost) on 19 patients is reported.

### Cavity volumes

**Figure 6:**
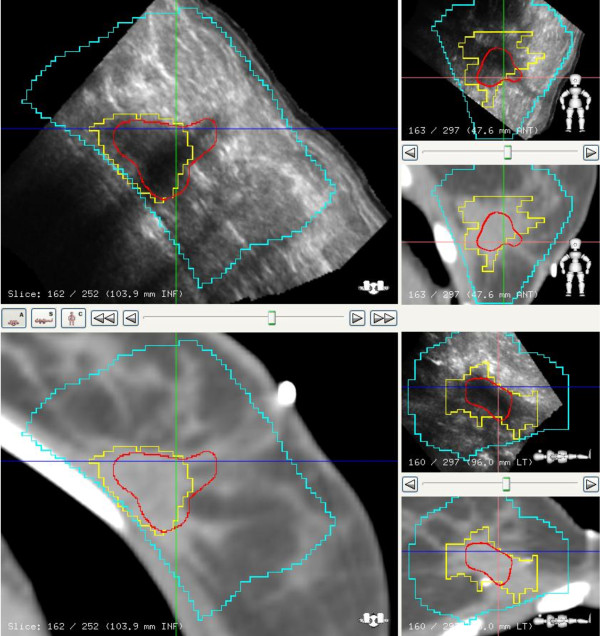
**CT image with clips, co-registered with US in axial, coronal and sagittal views**. CT cavity (GTV) is in yellow, US seroma (RV) is in red, and blue represents PTV. In this case the US provides additional information for seroma definition.

**Figure 7:**
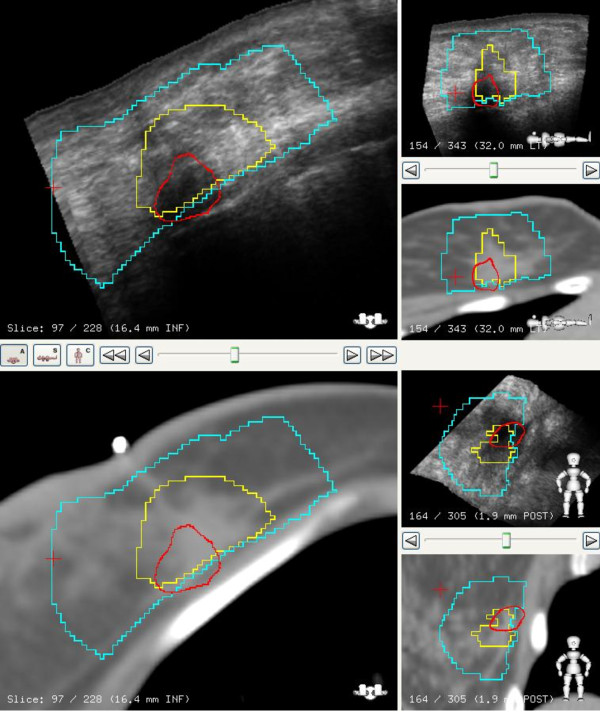
**US and CT image co-registration in axial, sagittal and coronal views**. The CT cavity (GTV) is in blue, US seroma (RV) in green, and the blue represents PTV. The RV is not always in the geometric center of the GTV.

The GTV-CT target volume in most cases was defined by surgical clips and seroma cavity identified on the CT image, Figure 6. The relationship between the GTV-CT and RV on US images showed that RV was smaller than the GTV-CT on average by 38% (SD 23%). Further, it was also noted that the RV-US was not always in the geometric center of the GTV-CT, Figure 7.

**Figure 8:**
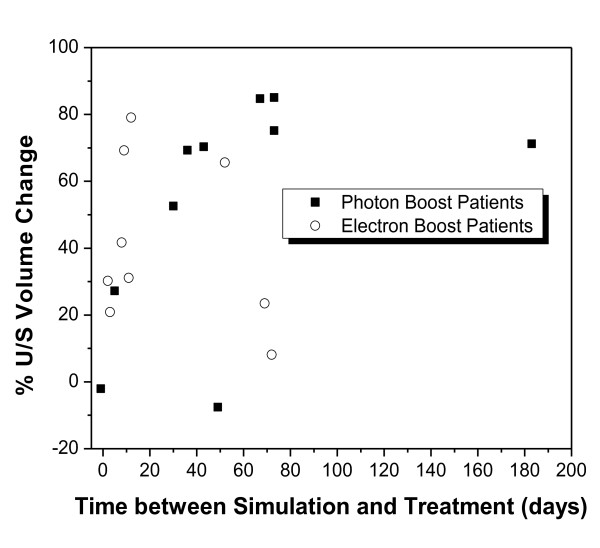
**Volume of seroma contoured on 3D-US at time of simulation (RV) and first RT fraction (SGV), for (a) photon boost, (b) electron boost**. Graph (c) plots percentage volume change over time between imaging sessions.

The average decrease in the RV during the time lapse between the simulation and the first boost treatment was noted to be 49% (SD 31%). The average time interval between the simulation and treatment session was 42 (SD 44) days. Volume change over elapsed time is shown in Figure 8.

### Photon boost fractions

**Figure 9:**
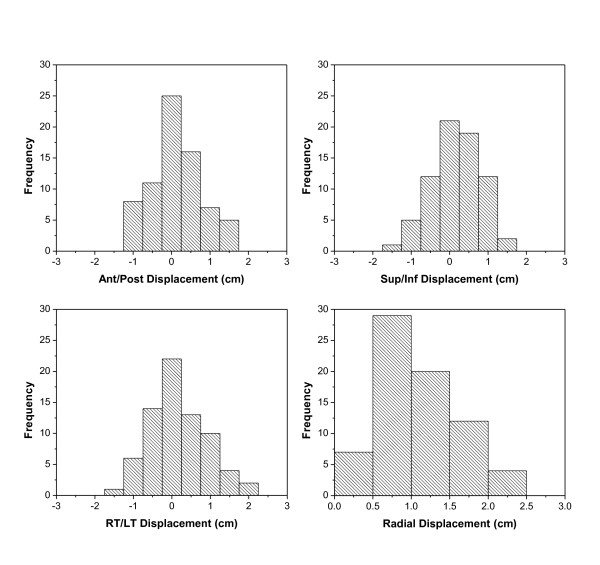
**Distribution of cavity shifts for photon boosts using (a) Ant/Post, (b) Sup/Inf, (c) Right/Left, (d) radial directions from the RV on the simulation US**.

**Figure 10:**
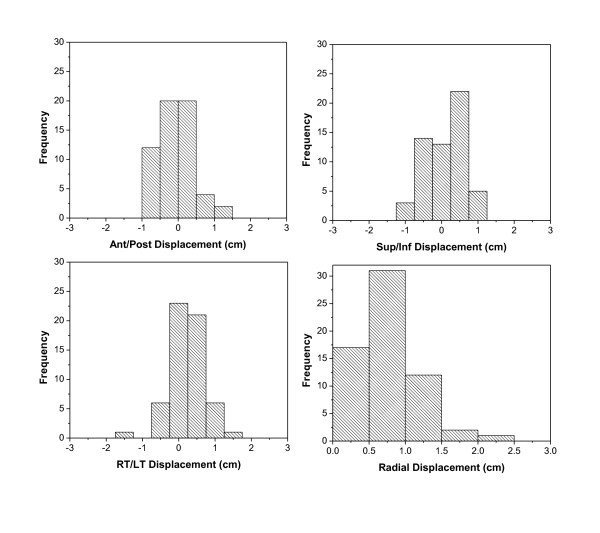
**Distribution of cavity shifts for photon boost in (a) Ant/Post, (b) Sup/Inf, (c) Right/Left and (d) radial directions, using GV from the first RT fraction US**.

Histograms of the IGRT shift (center of the GV to center of RV) are shown in Figure 9. The average radial shift was 1.1 ± 0.5 cm. Table 1. However, because we had also observed change in cavity volume between simulation and the first boost fraction, the magnitude of the shift could not entirely be attributed to variation in set up and motion of cavity during daily therapy. In order to exclude the effect of change in seroma volume used for image guidance during the boost phase, we evaluated the shifts of the GV between the first boost fraction and GV for the subsequent boost fractions. Histograms for these shifts excluding the RV are shown in Figure 10. The average radial shift was 0.8 ± 0.4 cm. Table 2.

**Table 1:** Average and standard deviation of photon boost cavity displacements in the three orthogonal directions, and total radial displacement

Displacement	R/L (cm)	S/I (cm)	A/P (cm)	Radial (cm)
Simulation US reference	0.2 ± 0.8	0.1 ± 0.7	0.2 ± 0.6	1.1 ± 0.5
1st Treatment Fraction US Reference	0.2 ± 0.5	-0.1 ± 0.5	0.1 ± 0.6	0.8 ± 0.4

Results are shown using both the simulation and first treatment fraction scans as the RV.

**Table 2:** Results of photon boost cavity displacements using both the simulation and first treatment fraction scans as the RV

	Simulation US Reference		1st Treatment Fraction US reference	
Radial Displacement (cm)	# fractions	Percentage of fractions	# fractions	Percentage of fractions
> 1.5	16	22%	3	5%
> 1.0	36	50%	15	24%
> 0.5	65	90%	46	73%

### Electron boost fractions

**Figure 11:**
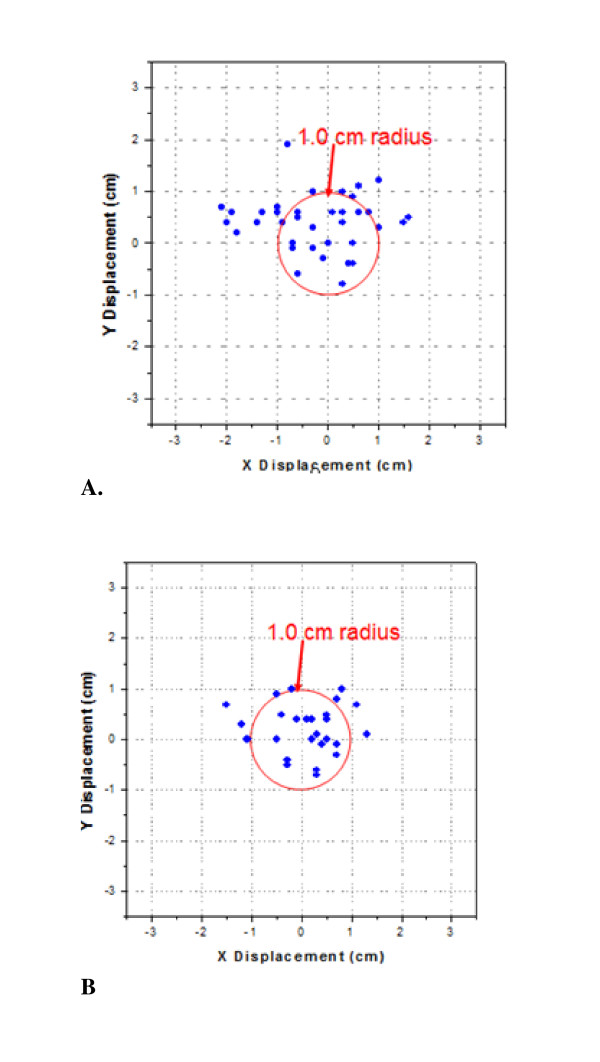
**Scatter plot of electron boost cavity displacements within the EBEV**: (a) using RV from simulation US, (b) using GV from the first RT fraction US.

For patients receiving electron boosts, using the RV from simulation US as reference to the GV during boost fractions we observed an average shift of 1.0 ± 0.5 cm, Table 3. Comparing GV from the first treatment fraction as reference for all subsequent fractions, the average shift was 0.8 ± 0.4 cm. The results of GV displacements in x and y collimator directions within 1 cm radius with reference to RV are projected in Figure 11a. This combined total EBEV shift is 1.0 ± 0.5 cm.

**Table 3:** Average and standard deviation of cavity displacements for e- boost in the x and y-collimator direction, and radial displacements, within the EBEV

Displacement	x (cm)	y (cm)	Total EBEV (cm)
Simulation US reference	-0.2 ± 1.0	0.4 ± 0.6	1.0 ± 0.5
1st Treatment Fraction US Reference	-0.1 ± 0.7	0.2 ± 0.5	0.8 ± 0.4

Results are shown using both the simulation and first treatment fraction scans as the RV.

The results for the cavity displacements between the GV of the first and subsequent treatment fractions are shown in Figure 11b. Smaller, yet clinically significant, IGRT shifts were identified with an average total shift of 0.8 ± 0.4 cm. Average and standard deviations of shifts in each direction are summarized in Table 4. We observed in 52% of fractions shifts > 1.0 cm using the RV from simulation, which decreased to 36% when the first fraction GV was used as reference.

**Table 4:** Cavity displacements for e-boost

	Simulation US Reference		1st Treatment Fraction US reference	
EBEV Displacement (cm)	# fractions	Percentage of fractions	# fractions	Percentage of fractions
> 1.5	8	26%	1	4%
> 1.0	16	52%	9	36%
> 0.5	26	84%	17	71%

Results are shown using both the simulation and first treatment fraction scans as the RV.

## Discussion

During the course of radiotherapy, there may be interfractional changes in the cavity position due to set-up, breast mobility, chest wall motion, and changes in cavity over time due to healing. In order to visualize the lumpectomy cavity for interfractional breast IGRT, some reports have utilized surface cameras for localization [19], which assumes that the cavity remains in a fixed position relative to the skin surface. X-ray modalities such as portal imaging or CBCT have limited or no ability to directly visualize the cavity, but can use surrogates such as surgical clips for this purpose [9,20,21]. Application of these technologies results in additional radiation exposure. Furthermore, there are conflicting results in the literature raising questions on the reliability of clips. Weed *et al *[9] studied the use of clips for IGRT and found that intrafractional cavity motion was clinically significant; Kim *et al *[20] suggest that clips are not an ideal surrogate for cavity localization. 3DUS gives a direct soft-tissue visualization of the cavity the image for 3DUS is target specific for visualization of the lumpectomy cavity and therefore optimal for IGRT in breast cancer. Furthermore, US technology is not associated with additional radiation exposure especially a consideration when daily imaging is required [22,23].

This study provided an opportunity to evaluate how best to apply 3D-US for IGRT. For an ultrasound image to be a specific and sensitive tool, visibility of the seroma is critical. In our study group, a 93% visibility of seroma makes the application of 3DUS guidance a practical tool for IGRT in breast cancer. These observations are comparable to reports by Wong *et al *and Berrang *et al *[10,11,18]. Although CT/3DUS fused datasets were not used for contouring GTV in this study, visual inspection of the fusion showed complementary information as suggested by others [18]. Further, we observed that the RV on US was always smaller and not always in the geometric center of the GTV-CT. In Figure 7, for example the US image illustrates seroma cavity protruding outside the GTV-CT contour, suggesting that fused dataset may enhance the accuracy for target delineation.

In this study, we exclusively contoured the seroma cavity on the 3D-US since our primary intent was to use the structure as a landmark for IGRT positioning purposes rather than treatment planning. We found that the seroma outlines gave the clearest and most reproducible edges to calculate daily IGRT shifts from simulation and treatment 3D-US images. This method effectively uses the seroma cavity imaged with 3D-US more as a fiducial guide for the target in performing IGRT rather than representing the target itself.

Our observations also helped define the importance of the timeline in which the images for IGRT are obtained. In this study, we acquired simulation data for the boost IGRT treatments only once using our standard practice of dosimetry planning on the one initial CT and 3D-US simulation. We observed a reduction of the cavity volume on 3DUS of 49% (SD 31) between the simulation and the first boost fraction. Further, this raises a question on how to accurately align the GV acquired at the time of treatment to the RV from simulation for IGRT. Intuitively, shifting the patient to center the GV on the RV may not ensure accurate coverage particularly if there has been asymmetric cavity shrinking. To circumvent this problem, we recommend the implementation of CT/3D-US simulation session just prior to the treatments that will use IGRT. Based on our observations we suggest that a second CT and 3D-US simulation should be performed just prior to starting the boost. This would help eliminate the variable of change in cavity volume and improve the accuracy of IGRT. Although a second CT/3D-US was not performed in this initial study, we simulated its effect by using the first boost fraction contour as the RV, and still observed shifts required to overlay the seroma volume during daily positioning. This observation suggests the potential value for IGRT in treatment of breast cancer.

Electron boosts, although physically similar in terms of interfractional cavity changes as noted by the similar shift results found in this study, are inherently different due to the nature of electron treatment setups and electron dose deposition. Shifts were compared to the edges of the electron cut-out, but electron doses can taper in significantly from these edges, depending on the electron energy. We primarily analyzed the displacements in the plane of the EBEV for this purpose. The average electron penumbra requires the target to be positioned within 1 cm to avoid target misses. Using the simulation RV as reference, 52% of fractions would have fallen outside of this range. Again, similar to the photon data review to simulate planning images acquired just prior to the boost, we compared the shifts using the GV from the first treatment fraction as reference for all subsequent fractions of GV. We still noted that 36% would have fallen outside of this range. This suggests that IGRT corrections would be beneficial for electron boost targeting with currently used cutout margins. IGRT may also allow use of more conformal fields as the physical margin for day-to-day positioning can be eliminated and smaller volumes are associated with better tolerance and lesser toxicity. It should be noted that with electron boost IGRT, an additional concern is maintaining the correct source-to-surface distance, as well as targeting surface landmarks such as the surgical scar. This can be accomplished with the Clarity system since landmarks are digitized with the pointer tool, and targeting adjustments can be accomplished while maintaining the prescribed SSD.

The dose response relationship in breast cancer has been illustrated through the boost vs. no boost randomized trial [24], and is known to significantly decrease the risk of local recurrence [25-27]. As demonstrated in this preliminary study, IGRT in breast cancer therapy has the potential for improving the accuracy of targeting the lumpectomy site. IGRT may also significantly improve the delivery of conformal partial breast irradiation, a treatment strategy currently being studied as an alternative to whole breast irradiation [28], as well as whole breast fractions for patients with cavities close to the tangent field edges.

## Conclusion

Our experience suggests that the Clarity Breast System can be used without significantly interfering with patient flow in a busy department. The presence of a seroma is noted in a relatively high percentage of patients even with long time intervals after the most recent surgery. The Clarity Breast System can successfully locate the seroma on a daily basis, without extra radiation exposure. For more accurate target volume delineation, data from both US and CT images should be used when contouring. The daily set-up isocenter shifts we observed suggests an opportunity for improving precision of RT dose targeting with IGRT. In order to use the seroma cavity for IGRT, the time interval between simulation and treatment should be kept at a minimum. Future work will include a second CT/3D-US simulation just prior to initiating IGRT treatment. Further study is needed to establish the optimal protocol for clinical application of 3D-US IGRT including defining the ideal times for image acquisition, optimal volume used for image guidance, and establish the clinical significance of improved targeting by correcting the interfractional shifts.

## Conflict of Interest

The authors have no conflict of interest

## Authors' contributions

MC, LH conceived of the study, and participated in its design and coordination. AY participated in the coordination and statistical analysis. CG and RM performed data acquisition on all images and contributed to the statistical analysis. All authors have approved the final manuscript.
